# Dietary supplementation with shiikuwasha extract attenuates dexamethasone-induced skeletal muscle atrophy in aged rats

**DOI:** 10.1186/s40064-016-2427-7

**Published:** 2016-06-21

**Authors:** Yasuyuki Sakata, Tomoyuki Okamoto, Kazutaka Oshio, Hirohiko Nakamura, Hiroshi Iwamoto, Kazuyoshi Namba, Yasuhiro Takeda, Fumiaki Yoshizawa

**Affiliations:** Nutrition Research Department, Nutritional Science Institute, Morinaga Milk Industry Co., Ltd., 1-83, 5-Chome, Higashihara, Zama, Kanagawa 252-8583 Japan; Department of Agrobiology and Bioresources, Faculty of Agriculture, Utsunomiya University, 350, Mine-machi, Utsunomiya, Tochigi 321-8505 Japan

**Keywords:** Rat, Skeletal muscle atrophy, Glucocorticoid, Shiikuwasha, Polymethoxylated flavones

## Abstract

**Background:**

Skeletal muscle atrophy is caused by a variety of diseases and conditions. In particular, skeletal muscle atrophy in the elderly contributes to a loss of independence with advanced age and increases the risk of falling. However, the effect of food consumed on a daily basis on skeletal muscle atrophy has been the focus of little research. In this study, the effects of dietary supplementation with shiikuwasha extract or grape extract on dexamethasone-induced skeletal muscle atrophy were evaluated in aged rats.

**Methods:**

Aged male rats (15-month-old) were fed a diet supplemented with either 1 % shiikuwasha extract or 1 % grape extract for 19 days. During the last 5 days of the feeding period, rats were injected with dexamethasone to induce muscle atrophy.

**Results:**

Body weight and hind-limb muscle weight were significantly decreased by dexamethasone treatment. The supplementation of shiikuwasha extract showed no effect on body weight loss, but markedly attenuated tibialis anterior muscle weight loss induced by dexamethasone. On the other hand, grape extract did not affect muscle weight loss. Furthermore, shiikuwasha extract significantly reduced dexamethasone-induced expression of atrogin-1 and MuRF1 mRNA, but did not reduce LC3B-II protein levels.

**Conclusion:**

These results suggest that shiikuwasha extract may partially inhibit the activation of the ubiquitin–proteasome system and may consequently attenuate skeletal muscle atrophy induced by dexamethasone in aged rats.

## Background

Skeletal muscle atrophy is caused by a variety of diseases and conditions including malnutrition, disuse, sepsis, cachexia, and glucocorticoid excess. In particular, skeletal muscle atrophy in the elderly contributes to a loss of independence with advanced age and increases the risk of falling (Landi et al. 2012); therefore, skeletal muscle atrophy is a very important problem in an aging society. Many pathological conditions that cause muscle atrophy, such as sepsis, cachexia and starvation, are associated with increases in circulating glucocorticoid levels (Schakman et al. 2008). Moreover, sepsis-induced muscle atrophy is attenuated by treatment with a glucocorticoid receptor antagonist (Fischer et al. 2000). These results suggest that glucocorticoids are partly responsible for muscle atrophy under these conditions. In addition, muscle atrophy induced by disuse is exacerbated by hypercortisolemia (Fitts et al. 2007); therefore, it is thought that many factors are involved in the progression of muscle atrophy.

Glucocorticoids such as dexamethasone induce body weight loss, skeletal muscle atrophy, and acute insulin resistance (Pagano et al. 1983; Ruzzin et al. 2005). In skeletal muscle, glucocorticoids induce selective loss of fast-twitch type-2 muscle fibers, and might not affect type-1 muscle fibers (Dekhuijzen et al. 1995). Ubiquitin- proteasome system and autophagy- lysosomal system play significant role in the protein degradation of muscle (Sandri 2013). It has been reported that both pathways are also activated during skeletal muscle atrophy induced by glucocorticoids, showing increased mRNA expression of muscle-specific ubiquitin E3 ligases, atrogin-1 and MuRF1 (Auclair et al. 1997), and the lipidated form of LC3 (LC3-II) protein, which is known as marker of autophagy (Penna et al. 2013). Glucocorticoids also affect muscle protein synthesis. Glucocorticoids reduce mRNA expression of IGF-1, which promotes protein synthesis (Gayan-Ramirez et al. 1999), and phosphorylated ribosomal protein S6 kinase 1 (S6K1), which is one of the regulators involved in the initiation of mRNA translation, in type-2 fiber rich muscles (Shimizu et al. 2011). The responsiveness of muscle protein metabolism to glucocorticoids is known to be affected by age. Muscle protein synthesis stimulated by insulin or IGF-1 is inhibited by glucocorticoids more strongly in aged rats than in young rats (Dardevet et al. 1998). In addition, glucocorticoid excess was observed to induce a prolonged leucine resistance in muscle protein synthesis in aged rats (Rieu et al. 2004). These results indicate that aging increases the sensitivity to glucocorticoid-induced muscle atrophy.

Polyphenols, widely contained in fruits, have been reported to have various physiological effects. Shiikuwasha (*Citrus depressa* Hayata) fruit contains many polyphenols, such as polymethoxylated flavones (Nogata et al. 2006). Nobiletin (5,6,7,8,3′,4′-hexamethoxy flavone), a polymethoxylated flavone from citrus, and its demethylated metabolites have been reported to have many beneficial effects, such as reduced oxidative stress (Lu et al. 2010), improved insulin sensitivity (Lee et al. 2010) and anti-inflammation (Lai et al. 2008). Grapes (*Vitis vinifera*) are also known to contain many polyphenols, including the stilbenoid resveratrol (Siemann and Creasy 1992). Resveratrol has been reported to have many functions, including SIRT1 activation (Howitz et al. 2003), anti-oxidant (Jackson et al. 2011) and anti-inflammation (Subbaramaiah et al. 1998) activities.

Interestingly, it has been reported that some polyphenols such as resveratrol and 8-prenylnaringenin are effective in preventing muscle atrophy caused by disuse or aging (Momken et al. 2011; Mukai et al. 2012). Moreover, resveratrol is reported to prevent dexamethasone-induced increases in protein degradation in cultured myotubes (Alamdari et al. 2012). However, the inhibitory effects of polyphenols on glucocorticoid-induced muscle atrophy in vivo have not been fully elucidated. The aim of this study was to examine whether extracts of these polyphenol-rich fruits have a protective effect on dexamethasone-induced muscle atrophy in aged rats.

## Methods

### Shiikuwasha and grape extracts

The shiikuwasha and grape extracts were obtained from ARKRAY, Inc. Karada Lab (Kyoto, Japan) and Oryza Oil & Fat Chemical (Aichi, Japan), respectively. Shiikuwasha extract contains cyclodextrin in order to increase its solubility in water. The polyphenols contained in these extracts are presented in Table 1.

**Table 1:** Composition of shiikuwasha (*Citrus depressa* Hayata) and grape (*Vitis vinifera*) extracts

Extract	Component	Proportion (%)
Shiikuwasha extract	Cyclodextrin	50.0
	Nobiletin (5,6,7,8,3′,4′-hexamethoxyflavone)	8.5
	Tangeretin (5,6,7,8,4′-pentamethoxyflavone)	4.1
	Other polyphenols	About 11.0
	Other ingredients	About 26.0
Grape extract	Resveratrol	5.0
	Other polyphenols	About 35.0
	Other ingredients	About 60.0

### Animals and treatment

Fifteen-month-old Sprague–Dawley rats were purchased from Charles River Japan (Yokohama, Japan). They were housed under controlled temperature (21–25 °C), humidity (40–60 %), and lighting (on at 8 a.m. and off at 8 p.m.). All animal studies were approved by the Animal Research Committee of Morinaga Milk Industry. Following 1 week of acclimatization, the rats were divided into 4 groups: control (CTL group; n = 6), dexamethasone (DEX group; n = 6), dexamethasone plus shiikuwasha extract (DEX + SE group; n = 5), and dexamethasone plus grape extract (DEX + GE group; n = 6). Shiikuwasha or grape extract was added to the standard AIN-93M rodent diet at 1 % by weight. Rats in the DEX + SE and DEX + GE groups were fed the diet supplemented with shiikuwasha or grape extract, respectively. CTL and DEX groups were fed AIN-93M purified rodent diet. Rats were fed each experimental diet for 19 days. To control for variations in dietary intake in the initial few days, after 1-week acclimatization to the experimental diets, dietary intake was recorded for the following 12 days. During the last 5 days of feeding, the DEX, DEX + SE and DEX + GE groups were injected with dexamethasone (750 μg/kg B.W.; Fujita Medical, Tokyo, Japan) intraperitoneally once per day between 10 and 11 a.m. The dose and treatment period of dexamethasone are based on the methods of Minet-Quinard et al. (2000). The CTL group was injected with an equal volume of saline. Rats in all groups, except the CTL group, were provided diet and water ad libitum. The CTL group was pair-fed with the DEX group during dexamethasone treatment. A day after the last dexamethasone treatment, the rats were anesthetized with sevoflurane and sacrificed. Immediately after, blood was taken and the hind limb muscles [gastrocnemius, soleus, tibialis anterior (TA), and extensor digitorum longus (EDL)] were excised. Blood samples were centrifuged at 1000*g* for 15 min, and sera were collected and stored at −80 °C until used in assays. The TA muscle was divided into two parts; one part of the TA and the EDL were frozen in liquid nitrogen, and the other part was stored in RNAlater (Applied Biosystems, Foster City, CA) at 4 °C overnight and then stored at −80 °C until analysis. Gastrocnemius muscles were excised, immediately weighed, and homogenized with 7 volumes of homogenization buffer (20 mM *N*-2-hydroxyethylpiperazine-*N*′-2-ethanesulforic acid at pH 7.4, 100 mM KCl, 0.2 mM EDTA, 2 mM ethylene glycol-bis (β-aminoethylether)-*N*,*N*,*N*′,*N*′,-tetraacetic acid, 1 mM dithiothreitol, 50 mM sodium fluoride, 50 mM β-glycerophosphate, 0.1 mM phenylmethyl sulfonyl fluoride, 1 mM benzamidine, and 0.5 mM sodium vanadate) using a Polytron homogenizer (Kinematica, Littau, Switzerland).

### Immunoblotting

The gastrocnemius muscle homogenate was centrifuged at 10,000*g* for 10 min at 4 °C. The supernatant was mixed with an equal volume of 2 × SDS sample buffer, and the diluted gastrocnemius muscle sample was subjected to electrophoresis on a 7.5 % polyacrylamide gel for S6K1 analysis or a 4–15 % gradient gel (Bio-Rad, Hercules, CA) for LC3B and GAPDH analyses. The samples were subjected to protein immunoblot analysis using S6K1 polyclonal antibodies (dilution 1:500, sc-230, Santa Cruz Biotechnology, Santa Cruz, CA), LC3B antibody (#2775, Cell Signaling Technology, Danvers, MA) or GAPDH antibody (#2775, Cell Signaling Technology). The S6K1 phosphorylation ratio was quantified using the ratio of the heavier phosphorylated forms (β + γ forms) to total immune reactivity (α + β + γ forms), because S6K1 resolves into multiple electrophoretic forms as a result of reduced electrophoretic mobility with increased phosphorylation, as described previously (Kimball et al. 1998, Yoshizawa et al. 2001). The protein content was normalized to GAPDH in the LC3B analysis.

### Protein content and TBARS

Protein concentrations of TA muscles were measured using the DC protein assay (Bio-Rad). The total protein content of each muscle was then calculated by multiplying protein concentration, homogenate volume, and the fraction of the ground portion relative to total muscle wet weight. TBARS was measured in whole EDL muscle homogenate by using the modified methods of Ohkawa et al. (1979).

### Serum insulin

Serum insulin concentrations were measured using a commercial ELISA kit (Morinaga Institute of Biological Science, Kanagawa, Japan).

### mRNA levels

Total mRNA of TA muscles was extracted with TRIzol reagent (Invitrogen, Carlsbad, CA) according to the manufacturer’s instructions. The isolated RNA was purified with an RNeasy mini kit (Qiagen, Dusseldorf, Germany), and its quality was analyzed using the Experion system (Bio-Rad). Total RNA was reverse-transcribed to cDNA using Taqman reverse transcription reagents (Applied Biosystems). Real-time PCR was performed with an ABI PRISM 7500 fast real-time PCR system (Applied Biosystems). The PCR primers and probes for atrogin-1 (Rn00591730), MuRF1 (Rn00590197), IGF-1 (Rn00710306) and GAPDH (Rn01775763) were purchased from Applied Biosystems. The target gene expression was normalized to the expression of the control gene GAPDH.

### Statistical analysis

All values are presented as mean ± SEM. JMP software (SAS Institute Inc., Cary, NC) was used for all statistical analyses. Results showing significant differences between the CTL and DEX groups, according to the Student’s *t* test, were analyzed with Dunnett’s test for multiple comparisons between the extract supplemented groups and the DEX group. Values of *P* < 0.05 were considered to be statistically significant.

## Results

### Body weight, dietary intake, and serum insulin

Body weights before dexamethasone treatment were not different among the groups (Table 2). During the dexamethasone treatment period, dietary intake in the DEX group was not different from that in the CTL group; however, body weight in the DEX group was significantly lower than in the CTL group. Body weight loss in both extract-supplemented groups (DEX + SE and DEX + GE) was not significantly different from that in the DEX group. Hyperinsulinemia was observed in the dexamethasone treated groups. A significant difference in serum insulin levels was not observed between either of the extract supplemented groups and the DEX group (Table 2).

**Table 2:** Effects of shiikuwasha or grape extract supplementation on dexamethasone-induced changes

	CTL	DEX	DEX + SE	DEX + GE
Body weight (g)				
Before DEX treatment	766.5 ± 32.8	757.6 ± 25.1	757.2 ± 20.5	777.0 ± 46.1
After DEX treatment	735.0 ± 31.9	638.5 ± 20.1*	654.5 ± 19.8	663.5 ± 37.6
Body weight loss (g)	31.57 ± 2.84	119.06 ± 8.68*	102.74 ± 10.66	113.58 ± 10.68
Average dietary intake (g)				
7 days before DEX treatment	22.4 ± 0.30	21.6 ± 0.72	19.4 ± 1.01	20.94 ± 0.51
5 days during DEX treatment	13.1 ± 0.14	11.52 ± 0.91	9.33 ± 0.86	11.14 ± 1.12
Serum insulin (ng/ml)	0.56 ± 0.13	12.03 ± 2.54*	8.42 ± 1.42	14.06 ± 2.98

Values are mean ± SEM (n = 5 or 6)

*CTL* control group, *DEX* dexamethasone treatment group, *DEX* + *SE* dexamethasone treatment and shiikuwasha extract supplemented group, *DEX* + *GE* dexamethasone treatment and grape extract supplemented group

* *P* < 0.05 CTL versus DEX (student t-test)

### Skeletal muscle weight

**Fig. 1:**
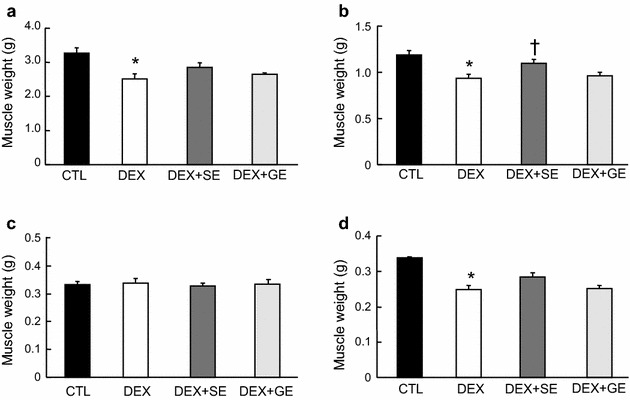
Effect of shiikuwasha extract or grape extract supplementation on dexamethasone-induced changes in hind-limb muscle weight. Rats were fed an extract-supplemented diet for 19 days. During the last 5 days of feeding, the DEX, DEX + SE and DEX + GE groups were injected with dexamethasone, while the CTL group was injected with an equal volume of saline. After 5 days of dexamethasone treatment, hind-limb muscles were collected and weighed. **a** Gastrocnemius muscle, **b** tibialis anterior muscle, **c** soleus muscle, **d** extensor digitorum longus muscle. *CTL* control, *DEX* dexamethasone, *DEX* + *SE* dexamethasone + shiikuwasha extract, *DEX* + *GE* dexamethasone + grape extract. Values are mean ± SEM (n = 5 or 6). *P < 0.05 CTL versus DEX (student’s t-test), ^†^ *P* < 0.05 versus DEX (Dunnett’s test)

The weights of the gastrocnemius, TA and EDL muscles were significantly reduced in the DEX group compared to the CTL group, while the weight of the soleus muscle was not changed (Fig. 1). TA muscle weight in the DEX + SE group was markedly higher than in the DEX group (Fig. 1b). Similarly, the EDL muscle weight tended to be increased in the DEX + SE group compared with the DEX group; however, this result was not statistically significant (*P* = 0.076; Fig. 1d). Furthermore, there was no difference in gastrocnemius muscle weight between the DEX group and the DEX + SE group (*P* = 0.107; Fig. 1a). There was no significant difference in the weights of each muscle between the DEX and DEX + GE groups.

### Muscle protein content

**Fig. 2:**
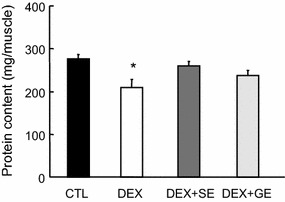
Effect of shiikuwasha extract or grape extract supplementation on dexamethasone-induced changes in protein content. Protein content of TA muscles was determined using the DC protein assay. *CTL* control, *DEX* dexamethasone, *DEX* + *SE* dexamethasone + shiikuwasha extract, *DEX* + *GE* dexamethasone + grape extract. Values are mean ± SEM (n = 5 or 6). *P < 0.05 CTL versus DEX (student’s t-test)

The protein content of the TA muscle in the DEX group was lower than in the CTL group (Fig. 2), while that in the DEX + SE group was higher than in the DEX group; however, this difference was not significant (*P* = 0.068). No difference was observed in protein content between the DEX and DEX + GE groups.

### Phosphorylation of S6K1 and lipidation of LC3B

**Fig. 3:**
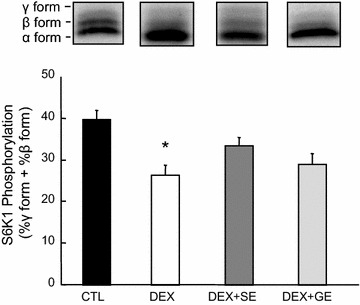
Effect of shiikuwasha extract or grape extract supplementation on dexamethasone-induced changes in S6K1 phosphorylation. Phosphorylation of S6K1 in gastrocnemius muscle was expressed as the amount of S6K1 in the β and γ forms as percentage of total S6K1. *CTL* control, *DEX* dexamethasone, *DEX* + *SE* dexamethasone + shiikuwasha extract, *DEX* + *GE* dexamethasone + grape extract. Values are mean ± SEM (n = 5 or 6). *P < 0.05 CTL versus DEX (student’s t-test)

**Fig. 4:**
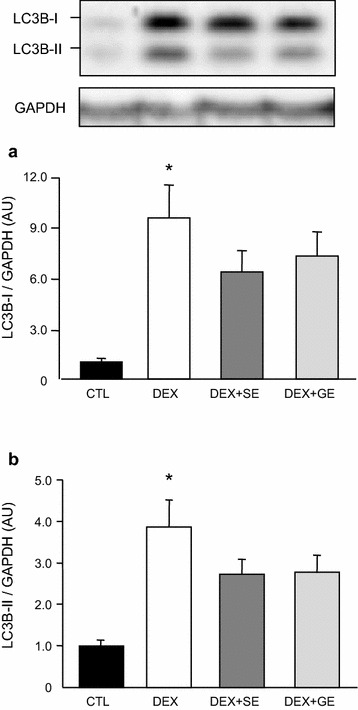
Effect of shiikuwasha extract or grape extract supplementation on dexamethasone-induced changes in LC3B-I and LC3B-II. Both LC3B-I and LC3B-II protein levels in gastrocnemius muscle were normalized to GAPDH. **a** LC3B-I normalized to GAPDH, **b** LC3B-II normalized to GAPDH. *CTL* control, *DEX* dexamethasone, *DEX* + *SE* dexamethasone + shiikuwasha extract, *DEX* + *GE* dexamethasone + grape extract. Values are mean ± SEM (n = 5 or 6). *P < 0.05 CTL versus DEX (student’s t-test)

The S6K1 phosphorylation ratio, which is calculated by dividing β + γ forms by total forms, in gastrocnemius muscle was markedly lower in the DEX group than in the CTL group (Fig. 3). Although S6K1 phosphorylation in the DEX + SE group was marginally higher than in the DEX group, the difference was not statistically significant (*P* = 0.122). There was no difference in S6K1 phosphorylation level between the DEX and DEX + GE groups. Both LC3B-I and LC3B-II levels in gastrocnemius muscle were significantly increased by dexamethasone treatment (Fig. 4). Although LC3B-II protein levels in the DEX + SE and DEX + GE groups were slightly lower than in the DEX group, the differences were not significant (*P* = 0.163, 0.162 respectively).

### mRNA expression

**Fig. 5:**
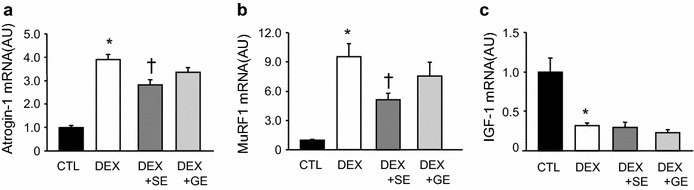
Effect of shiikuwasha extract or grape extract supplementation on dexamethasone-induced changes in mRNA expression. The mRNA was extracted from tibialis anterior muscles and measured using RT-PCR. The mRNA expression was normalized to GAPDH. **a** Atrogin-1 mRNA expression, **b** MuRF1 mRNA expression, **c** IGF-1 mRNA expression. *CTL* control, *DEX* dexamethasone, *DEX* + *SE* dexamethasone + shiikuwasha extract, *DEX* + *GE* dexamethasone + grape extract. Values are mean ± SEM (n = 5 or 6). *P < 0.05 CTL versus DEX (student’s t-test), ^†^ *P* < 0.05 versus DEX (Dunnett’s test)

In the TA muscle, atrogin-1 (Fig. 5a) and MuRF1 (Fig. 5b) mRNA were significantly increased, while IGF-1 mRNA (Fig. 5c) was significantly decreased following dexamethasone treatment. Atrogin-1 (Fig. 5a) and MuRF1 (Fig. 5b) mRNA expression in the DEX + SE group was significantly lower than in the DEX group; however, there was no difference between the DEX + GE and DEX groups. No difference in IGF-1 mRNA expression was detected between the DEX group and either of the extract supplemented groups (Fig. 5c).

### Thiobarbituric acid reactive substances

Muscle oxidative stress was assessed by measuring thiobarbituric acid reactive substances (TBARS). There were no significant differences in TBARS levels among all groups (CTL: 1.92 ± 0.43, DEX: 2.13 ± 0.37, DEX + SE: 2.30 ± 0.48, DEX + GE: 1.21 ± 0.09 μmol MDA/g protein).

## Discussion

In this study, we examined the inhibitory effects of polyphenol-rich fruit extracts on skeletal muscle atrophy. The supplementation of shiikuwasha extract significantly attenuated dexamethasone-induced weight loss in TA muscle (*P* = 0.040) and tended to attenuate weight loss in EDL muscle (*P* = 0.076). To take into account the water and fat contents in muscle weight, we measured the protein content of muscle. The dexamethasone-induced decrease in TA protein content tended to be attenuated by the diet supplemented with shiikuwasha extract (*P* = 0.068). Grape extract, on the other hand, did not have any noteworthy effect on muscle atrophy.

The catabolic action of glucocorticoids on skeletal muscle is thought to result from the activation of proteolytic pathways, in particular the ubiquitin–proteasome system and the autophagy-lysosomal system. It has been reported that glucocorticoids activate FOXO and KLF15 expression via the glucocorticoid receptor (GR) (Shimizu et al. 2011). These proteins promote gene expression of atrophy-stimulating genes such as atrogin-1 and MuRF1. In the present study, the supplementation of shiikuwasha extract reduced dexamethasone-induced increases in atrogin-1 and MuRF1 mRNA expression. Although we did not examine the effects of shiikuwasha extract on FOXO and KLF15, it is possible that shiikuwasha extract also inhibits the expression and/or activation of these factors. Penna et al. reported that dexamethasone treatment elevated LC3B-I and LC3B-II protein levels and reduced P62/SQSTM1 levels in young mice (Penna et al. 2013). In this study, LC3B-I and LC3B-II were increased similarly by dexamethasone treatment. It has been suggested that autophagy is activated by dexamethasone administration. However, the supplementation of shiikuwasha extract did not reduce LCB3-I and LC3B-II protein levels. These results indicate that shiikuwasha extract may inhibit the elevation of the ubiquitin–proteasome system but does not affect the autophagy-lysosomal system in glucocorticoid-induced muscle atrophy.

Glucocorticoids also inhibit muscle protein synthesis. Phosphorylation of S6K1 and eIF4E-binding protein 1 (4E-BP1) are inhibited by glucocorticoids via blunting of anabolic stimuli such as insulin, insulin-like growth factor-I (IGF-I), and amino acids (leucine) (Shah et al. 2000a, b; Liu et al. 2001, 2004). In the present study, S6K1 phosphorylation was significantly reduced by dexamethasone treatment. The supplementation of shiikuwasha extract slightly attenuated the inhibition of S6K1 phosphorylation, while the supplementation of grape extract had no effect. In addition, IGF-1 mRNA expression was significantly reduced by dexamethasone treatment. Neither extract had any effect on IGF-1 mRNA expression. There are a few reports concerning the influence of nobiletin on protein synthesis. Lee et al. reported that nobiletin upregulates Akt phosphorylation in diabetic ob/ob mice (Lee et al. 2010). This result suggests that polymethoxylated flavones improve protein synthesis under certain catabolic conditions, such as diabetes mellitus. Similarly, shiikuwasha extract may also have a positive effect on mTOR signaling and protein synthesis.

Glucocorticoid excess induces acute insulin resistance (Pagano et al. 1983) and increases oxidative stress (Iuchi et al. 2003). Antioxidant vitamins C and E have been reported to improve glucocorticoid-induced glucose intolerance (Williams et al. 2012). Furthermore, vitamin E attenuates oxidative stress and skeletal muscle atrophy induced by glucocorticoids (Ohtsuka et al. 1998). One possible mechanism of these beneficial effects of vitamins E and C could be due to a reduction in glucocorticoid-induced oxidative stress. In this study, serum insulin was increased by dexamethasone, however TBARS in the EDL muscle was not increased. The reason why an increase in the TBARS level was not observed is probably due to the elevation of TBARS with increasing age. Furthermore, dietary supplementation with either shiikuwasha or grape extract did not have an effect on the levels of TBARS and serum insulin. Decreased oxidative stress and increased endogenous anti-oxidative enzymes associated with the intake of polyphenols are suggested as plausible mechanisms by which polyphenols attenuate skeletal muscle atrophy. In particular, it has been indicated that resveratrol, via a pathway that involves the activation of SIRT1 and the up-regulation of antioxidant defense mechanisms (Ungvari et al. 2009), attenuates oxidative stress in aged skeletal muscle (Jackson et al. 2011). While an effect of dietary supplementation with shiikuwasha on TBARS levels was not observed in the present study, the possibility that shiikuwasha extract has an inhibitory effect on oxidative stress, and that this effect is involved in the attenuation of skeletal muscle atrophy, cannot be discounted. This contention is supported by the fact that shiikuwasha extract contains nobiletin, which has antioxidative activity (Lu et al. 2010), and by the observation that an increase in oxidative stress in response to dexamethasone was not observed in the present study. Many studies have provided evidence of the inhibitory effects of resveratrol on muscle atrophy in various animal models, such as disuse (Jackson et al. 2010), diabetes (Chen et al. 2011), and cancer cachexia (Shadfar et al. 2011). In addition, resveratrol inhibited dexamethasone-induced protein degradation in L6 myotubes (Alamdari et al. 2012); however, a grape extract containing resveratrol had no effect on skeletal muscle atrophy in the present study. Shadfar et al. suggested that a resveratrol dose of at least 200 mg/kg/day might be necessary to prevent cancer cachexia-induced muscle atrophy (Shadfar et al. 2011). The dose of grape extract used in this study may be insufficient to prevent glucocorticoid-induced muscle atrophy.

Neither the inhibitory effect of shiikuwasha extract nor that of polymethoxylated flavones in shiikuwasha fruit on muscle atrophy has been reported. In addition, there are few reports on the effects of this extract or polymethoxylated flavones on either protein synthesis or degradation. Further studies are needed to elucidate the mechanisms of the inhibitory effects of shiikuwasha extract on muscle atrophy. It is also necessary to determine the effect of shiikuwasha extract on muscle atrophy resulting from other causes such as disuse.

## Conclusions

We found that shiikuwasha extract attenuated dexamethasone-induced skeletal muscle atrophy in aged rats. Although the exact mechanism has not been fully defined, shiikuwasha extract may partially inhibit the activation of the ubiquitin–proteasome system and may consequently attenuate skeletal muscle atrophy induced by dexamethasone in aged rats. Our findings indicate that shiikuwasha extract could be useful for attenuating muscle atrophy associated with increases in circulating glucocorticoid levels, such as in sepsis, cachexia, and starvation.
